# Mean Platelet Volume and Splenomegaly as Useful Markers of Subclinical Activity in Egyptian Children with Familial Mediterranean Fever: A Cross-Sectional Study

**DOI:** 10.1155/2015/152616

**Published:** 2015-07-13

**Authors:** Huda Marzouk, Hala M. Lotfy, Yomna Farag, L. A. Rashed, Kamal El-Garf

**Affiliations:** ^1^Department of Pediatrics, Faculty of Medicine, Cairo University, Cairo 11562, Egypt; ^2^Department of Medical Biochemistry, Faculty of Medicine, Cairo University, Cairo 11562, Egypt; ^3^Department of Rheumatology, Faculty of Medicine, Cairo University, Cairo 11562, Egypt

## Abstract

*Objective*. To study whether mean platelet volume (MPV) and splenomegaly could be used as subclinical inflammatory markers in children with familial Mediterranean fever (FMF) at the attack-free period. *Patients and Methods*. The study included ninety-seven children with FMF. MPV was carried out within 4 hours of blood sampling according to standard laboratory practice. Splenomegaly was determined by abdominal ultrasound (USG). *Results*. High MPV was detected in 84.45% of our studied patients and was significantly higher in FMF patients with splenomegaly than in patients without splenomegaly. There was a statistically significant correlation between MPV and splenic span (*P* = 0.045). *Conclusion*. Elevated MPV and its significant correlation with splenic span in FMF children during the attack-free periods support the use of MPV and splenomegaly as useful markers of the subclinical inflammation in FMF patients at the attack-free period.

## 1. Introduction

Familial Mediterranean fever (FMF) is an autosomal recessive disorder characterized by recurrent attacks of peritonitis, arthritis, pleuritis, and erysipelas-like erythema [1]. The disease most commonly occurs in Turks, Armenians, Jews, and Arabs [2]. FMF is caused by Mediterranean fever gene mutation (MEFV); this gene was mapped to the short arm of chromosome 16 [3, 4].

The clinical attacks of FMF are associated with increased erythrocyte sedimentation rate (ESR), C-reactive protein (CRP), serum amyloid A (SAA), and fibrinogen [5]. All these laboratory parameters usually return to normal values in attack-free periods [5, 6]. In some FMF patients, subclinical inflammation is known to continue during the attack-free periods [7].

The spleen is a platelet reservoir; the splenomegaly may be related to increase in hemolysis or may be caused by vascular, infectious, infiltrative, or inflammatory disorders [8]. Affection of reticuloendothelial system in FMF was mentioned in few studies [8–10]; Dursun et al. [8] reported splenomegaly in 27.9% of studied FMF children during attack-free period and Aharoni et al. [9] found splenomegaly in 27.5% and 13.3% of FMF patients at attack and at attack-free period, respectively. In addition, Rimon et al. [10] were the first who diagnosed retroperitoneal lymphadenopathy in FMF patients during life, by means of abdominal ultrasound and computerized tomography (CT).

MPV is the volume of average circulating platelet in femtoliters (fL) and is correlated with platelet function and activation [11]. In recent years, MPV was studied as a simple inflammatory marker in many diseases. Increased MPV was reported in cerebrovascular diseases and myocardial infarction, while decreased MPV was detected in active rheumatoid arthritis [11–17]. In addition, MPV is proposed to be an indicator of possibility of atherosclerosis [18]. The measurement of MPV in FMF has been investigated in few studies [8, 17, 19–21].

The aim of the present study is to study whether MPV and splenomegaly could be used as markers of the subclinical inflammation in children with FMF at the attack-free periods.

## 2. Patients and Methods

The study enrolled 97 patients with FMF at the attack-free periods, diagnosed according to the new pediatric FMF criteria [22, 23]. The attack-free period was defined as at least 2 weeks from the end of the latest FMF attack according to physical examination and clinical symptoms. All patients included were followed up at the Pediatric Rheumatology Clinic of Cairo University Specialized Pediatric Hospital during the period from June 2011 to December 2013. The samples for MPV measurement were extracted from the patients at the same day of the evaluation of splenomegaly. Inclusion criteria for the included patients were age of disease onset before 18 years and all patients receiving colchicine. Patients who had hepatitis C virus, recent thrombotic episode, diabetes mellitus, uncontrolled hypertension, or abnormal urine analysis for proteinuria were excluded from the present study. Also, patients who had received anticoagulant therapy or nonsteroidal anti-inflammatory drugs (NSAIDs) for 2 weeks before the study were excluded.

For all patients, thorough history taking was performed, including demographic data, age at disease onset and diagnosis, the duration between disease onset and diagnosis, first presenting symptoms, and frequency and duration of attacks. Colchicine duration, adherence to colchicine (adherence was defined as no colchicine dose was missed in at least the last 6 months before the time of study), and the MEFV gene mutations of the patients were recorded from patients' files, together with full physical examination. Complete blood count (CBC), CRP, ESR, and urine analysis were measured by standard laboratory methods at the time of study.

Splenomegaly was determined by abdominal ultrasound (USG). In USG, splenic span was reported according to age and height standards for splenomegaly in children [24]. Patients had been classified according to the presence of splenomegaly or not into 2 groups, group 1 (patients with splenomegaly, *n* = 43) and group 2 (patients without splenomegaly, *n* = 54).

The study was approved by the Cairo University Clinical Research Ethics Committee, and informed consents were taken from the parents of all participants.

*MPV Measurement.* For MPV measurement, the blood samples were anticoagulated by ethylenediamine tetraacetic acid (EDTA) [25] and then treated with rapid processing within <4 hours. The measurement was done according the standard laboratory technique [25]. CBC was measured using a Coulter blood cell counter (Cell-Dyn 3700; Abbott), CRP was done using latex agglutination test, and the ordinary manual method of ESR measurement was used [26].

## 3. Statistical Analysis

Data analysis was performed through Statistical Package of Social Sciences (SPSS) software program for Windows version 21. Data was expressed as mean and standard deviation (if parametric) or median and percentiles (if nonparametric) for quantitative variables and number and percentage for qualitative one. Comparison was performed through Chi square or Fisher exact test for qualitative and independent sample *t*-test (if parametric) or Mann-Whitney test (if nonparametric) for quantitative variables. Pearson or Spearman correlation coefficients were calculated for association of different quantitative variables. *P* value less than 0.05 was considered significant.

## 4. Results

The study included 97 patients with FMF, 50 males and 47 females. Their mean age at disease onset was 4.7 ± 2.7 years. Family history of FMF was detected in 14.4% of patients and the parents of 32% of patients were consanguineous.

Demographic data were outlined in Table 1.

Clinical and laboratory characteristics of the studied patients were summarized in Table 2.

The most frequent symptom was abdominal pain, occurring in 96 patients (99%), followed by fever in 87 (89.7%), arthralgia in 77 (79.4%), chest pain in 67 (69.1%), myalgia in 38 (39.2%), arthritis in 32 (33%), and convulsion in 7 (7.2%).

Splenomegaly was detected in 43 patients (44.3%) and the mean of splenic span was 8.8 ± 1.6. MPV was higher than normal value in 82 patients (84.5%). Only 57.7% of the studied patients were adherent to colchicine. All patients had MEFV gene mutation.

Comparison between FMF patients with splenomegaly (group 1) and those without splenomegaly (group 2) was outlined in Table 2.

The duration between the onset of symptoms and diagnosis was prolonged in group 1 compared to in group 2 with significant difference between the 2 groups (*P* = 0.04); however, there was no significant difference between the 2 groups as regards mean age at disease onset (*P* = 0.4) and the mean age at diagnosis (*P* = 0.7). The mean duration of attacks in group 1 and group 2 was 2.14 ± 2.07 and 1.43 ± 1.50 days, respectively, with significant difference (*P* = 0.02), while there was no significant difference between both as regards frequency of attacks/month (*P* = 0.7). There were no significant differences between group 1 and group 2 as regards mean duration of colchicine administration and the adherence to colchicine (*P* = 0.6 and 0.1, resp.). There was no statistically significant difference between the 2 groups as regards the type of gene mutation.

The MPV values were statistically significantly higher in group 1 (9.01 ± 2.37) than in group 2 (7.48 ± 1.94), *P* = 0.001. There was no significant difference between the 2 groups as regards platelet count, CRP titer, and ESR.

Convulsion was significantly frequent in group 1 compared to in group 2 (14% and 1.9%, resp., *P* = 0.04), while chest pain was significantly frequent in group 2 compared to in group 1 (45% and 22%, resp., *P* = 0.001).

Statistical correlations between MPV and variable parameters were summarized in Table 3.

**Figure 1:**
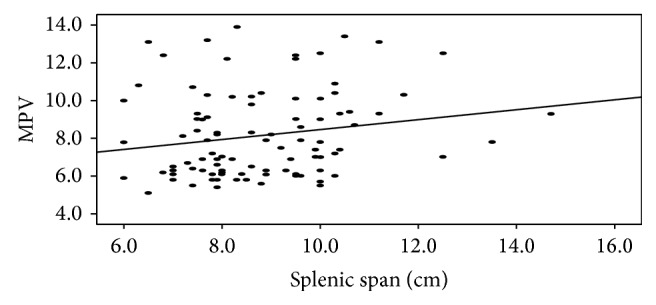
Correlation between MPV and splenomegaly.

Positive significant correlation was found between MPV and splenic span (*r* = 0.204, *P* = 0.045) (Figure 1). There was no significant correlation between MPV, CRP titer, ESR, platelet count, type of MEFV gene mutations, and colchicine treatment duration.

## 5. Discussion

Few studies investigated the involvement of reticuloendothelial system in FMF patients [8–10]. In the present study, 43 of the patients (44.3%) had splenomegaly at time of study. This was found to be more frequent than that reported by Dursun et al. [8] and Aharoni et al. [9] who reported splenomegaly in 27.9% and 13.3%, respectively, of FMF patients at attack-free periods. Discrepancy between the results may be related to variation in the study design, which also may be related to low adherence of our patients to colchicine treatment (only 57.7% were adherent).

In the present study, we did not find a correlation between splenomegaly and the type of gene mutation in FMF patients which is consistent with the result of Dursun et al. [8] and Inal et al. [27]. On the other hand, Paut et al. [28] found a significant correlation between splenomegaly and homozygosity for M694V mutation. The differences between the results may be related to ethnic difference and variation in the type of genetic mutation between Egyptian FMF patients and patients from other countries.

Recent studies showed that MPV may be used as a marker of inflammation and efficacy of treatment in several chronic inflammatory diseases [11–15, 29, 30]. In the present study, high MPV was detected in 84.5% of the FMF patients during the attack-free periods, similar to the results reported by Arica et al. [20] and Coban and Adanir [21]. On the contrary, Sahin et al. [17] reported that MPV levels were significantly low in FMF patients during either attack or attack-free periods. Many studies reported that some proinflammatory cytokines (one example to these cytokines is IL-6), which are elevated in FMF patients, might affect platelet volume by inducing thrombocytosis [31–33]. These cytokines may be even elevated during the subclinical inflammation at the attack-free periods in FMF patients, resulting in increased MPV and increased risk of atherosclerosis, as many authors reported that high MPV in FMF patients may result in the development of atherosclerosis [19, 34, 35].

Significant correlation between splenomegaly and MPV was found in the present work (*r* = 0.204, *P* = 0.045). To our current knowledge, there is only one study documenting this significant correlation in FMF children [8]. These results suggest the role of splenomegaly and MPV as a subclinical inflammatory marker in FMF children in between the attacks.

In the present study, no correlation was found between MPV and platelet count, which was similar to the result of Arica et al.'s [20] study in FMF patients during the attack-free periods. On the contrary, Makay et al. [19] reported correlation between MPV and platelet count. Also, we did not find correlation between MPV, ESR, and CRP titer similar to the results of Makay et al. [19]. These findings confirm the benefit of MPV as a marker of subclinical inflammation even in patients with normal ESR and CRP titer to allow early management and protect the FMF patients from the risk of developing complications especially amyloidosis and atherosclerosis.

We did not find a correlation between MPV and the duration of colchicine administration similar to what was reported by Arica et al. [20].

## 6. Conclusion

The present study demonstrated high MPV in 84.5% of studied FMF patients at the attack-free period. FMF children are susceptible to increased platelet activation and increased MPV values due to systemic inflammatory response of the disease even in attack-free periods due to subclinical inflammatory response.

The present study detected significant correlation between MPV and splenomegaly as a subclinical inflammatory marker in FMF children in attack-free periods. High percentage of FMF children with subclinical activity may be related to the low adherence of the studied children to colchicine.

Monitoring the FMF patients at the attack-free period using MPV together with splenomegaly enables us to detect subclinical inflammation and to adjust the lines of treatment in the FMF patients to avoid the possible complications. We recommend further studies with larger number of patients to compare MPV during and in between FMF attacks.

## Figures and Tables

**Table 1:** Characteristics of studied FMF patients (*n* = 97).

Variables	Frequency (%)/ mean ± SD
Male/female	50/47 (51.5/48.5)
Age at time of study (years)	8.7 ± 3.4
Age at disease onset (years)	4.7 ± 2.7
Age at disease diagnosis (years)	6.7 ± 3.2
Duration between the disease onset and diagnosis (years)	1.9 ± 2.0
Frequency of attacks/month	3.3 ± 2.9
Duration of attacks (days)	1.7 ± 1.8
Colchicine duration (years)	2.0 ± 1.9
Adherence to colchicine	56 (57.7)
MPV (fL)	8.2 ± 2.3
Splenic span (cm)	8.8 ± 1.6

Quantitative data are represented as mean ± standard deviation, while qualitative data are represented as frequency (%).

**Table 2:** Characteristics, clinical findings, and laboratory investigations of FMF patients stratified by the presence of splenomegaly (*n* = 97).

Variable	Patients with splenomegaly	Patients without splenomegaly	*P* value^*∗*^
	(*n* = 43)	(*n* = 54)	
Male/female	21/22	29/25	0.7
Positive consanguinity	16 (37.2%)	15 (27.8%)	0.4
Family history of FMF	7 (16.3%)	7 (13%)	0.8
Age at time of study	8.74 ± 3.59	8.68 ± 3.29	0.9
Age at disease onset	4.42 ± 2.81	4.92 ± 2.68	0.4
Age at diagnosis	6.81 ± 3.33	6.58 ± 3.03	0.7
Duration between the disease onset and diagnosis	2.25 ± 1.93	1.63 ± 2.07	**0.04**
Frequency of attacks/month	3.44 ± 3.00	3.18 ± 2.90	0.7
Duration of attacks/day	2.14 ± 2.07	1.43 ± 1.50	**0.02**
Colchicine duration (years)	2.06 ± 1.86	1.93 ± 1.96	0.6
Adherence to colchicine	21 (48.8%)	35 (64.8%)	0.1
Abdominal pain	42 (97.7%)	54 (100%)	0.4
Fever	37 (86%)	50 (92.6%)	0.3
Arthralgia	34 (79.1%)	43 (79.6)	1
Chest pain	22 (51.2%)	45 (83.3%)	**0.001**
Myalgia	18 (41.9%)	20 (37%)	0.7
Arthritis	17 (39.5%)	15 (27.8%)	0.3
Convulsion	6 (14%)	1 (1.9%)	**0.04**
MPV^a^ (fL)	9.01 ± 2.37	7.48 ± 1.94	**0.001**
ESR^b^ (mm/h)	29.37 ± 20.19	32.32 ± 19.59	0.4
CRP^c^ titer (mg/L)	13.77 ± 11.37	14.89 ± 13.28	0.5
Platelet count (×10^3^/mm^3^)	312.58 ± 75.34	327.32 ± 83.01	0.4
TLC^d^ (×10^3^/mm^3^)	7.37 ± 3.23	7.79 ± 3.11	0.5
V726A allelic	16 (18.6%)	16 (14.8%)	0.5
M694I allelic	10 (11.6%)	16 (14.8%)	0.5
E148Q allelic	8 (9.3%)	14 (12.9%)	0.5
M694V allelic	8 (9.3%)	13 (12%)	0.6
M680I allelic	7 (8.1%)	9 (8.3%)	1
P369S allelic	0	1 (0.9%)	1

Quantitative data are represented as mean ± standard deviation, while qualitative data are represented as frequency (%).

^*∗*^ *P* value < 0.05 is significant. ^a^MPV: mean platelet volume; ^b^ESR: erythrocyte sedimentation rate; ^c^CRP: C-reactive protein; ^d^TLC: total leucocytic count.

**Table 3:** Statistical correlations between MPV and variable parameters.

Variables	MPV^a^	
	*r*	*P* value^*∗*^
Age at time of study	0.001	0.995
Age at disease onset	−0.038	0.714
Age at disease diagnosis	−0.015	0.887
Duration between the disease onset and diagnosis	0.067	0.513
Disease duration (time from onset of symptom till now)	0.048	0.643
Frequency of attacks/month	−0.132	0.208
Duration of attack/day	−0.045	0.669
Colchicine duration (years)	0.144	0.163
Splenic span	**0.204**	**0.045**
ESR^b^	−0.009	0.932
CRP^c^ titer	0.163	0.427
Platelet count	0.107	0.296
TLC^d^	0.029	0.780

^*∗*^ *P* value < 0.05 is significant. ^a^MPV: mean platelet volume; ^b^ESR: erythrocyte sedimentation rate; ^c^CRP: C-reactive protein; ^d^TLC: total leucocytic count.
